# Variable Anatomy of the Middle Cerebral Artery from Its Origin to the Edge of the Sylvian Fissure: A Direct Fresh Brain Study

**DOI:** 10.1155/2021/6652676

**Published:** 2021-03-09

**Authors:** Eve M. Oo, Khin E. E. Saw, Hnin N. Oo, Thida Than, Khin Thida

**Affiliations:** ^1^Department of Anatomy, University of Medicine Taunggyi, Taunggyi 06017, Myanmar; ^2^Department of Anatomy, University of Medicine Magway, Magway 04011, Myanmar; ^3^Department of Anatomy, University of Medicine 1, Yangon 11131, Myanmar

## Abstract

The middle cerebral artery (MCA) is a major artery supplying blood to the brain and a common site of surgically treatable intracranial aneurysms. The MCA has anatomic variations that may have clinical significance. In order to investigate and document the extent of such variations, the MCA in 100 fresh brain hemispheres from 50 deceased patients, obtained from the Police Surgeon Office, Yangon General Hospital, Myanmar, was dissected and examined. Double MCA was observed in 2% of specimens. The termination patterns were bifurcation (72%), trifurcation (16%), and primary trunk (12%); early bifurcation was also observed (3%). The mean length of the main trunk (MT) was 20.6 ± 6.2 mm. The number of perforators ranged from 4 to 15 (mean = 9); most arose from the MT (96%), and the others originated at the bifurcation point (3%) and in postbifurcation divisions (1%). All of the perforators (100%) had a single branching pattern. The number of cortical branches ranged from 6 to 13 and included the orbitofrontal (98%), prefrontal (99%), precentral (95%), central (98%), temporopolar (87%), anterior temporal (89%), middle temporal (24%), posterior temporal (62%), temporo-occipital (69%), anterior parietal (88%), angular (83%), and posterior parietal (57%) arteries. Early cortical branches emerged from the MT in 52% of specimens. These data can help anatomists, radiologists, and neurosurgeons in preoperative assessment, surgical planning, and selection of surgical approach.

## 1. Introduction

Although it is generally assumed that human anatomy is uniform across individuals, many intraspecies differences exist. Anatomic variations have been extensively described and continue to be reported as incidental findings. The frequency and extent of variations depend on the specific population as they are mainly determined by genetic factors. Despite being largely asymptomatic, such variations pose diagnostic and therapeutic challenges in routine clinical procedures and have broad clinical significance [1–3].

The middle cerebral artery (MCA) is the largest, most important, and most complex of the cerebral vessels and supplies blood to many cortical areas and deep brain structures. Variations in its numbers influence coverage area, resulting in distinct clinical manifestations. Anatomic variations of the MCA have been linked to neurovascular diseases; for example, increased hemodynamic stress caused by structural irregularities may predispose to saccular aneurysms [4–9]. Computed tomography angiography is used for initial evaluations of the cerebral circulation in the context of neurologic disorders, and many studies have reported MCA variations as a radiologic reference [10].

MCA variations have clinical significance on multiple levels. For example, they may predispose to certain diseases or influence clinical presentation. Moreover, they can guide neurosurgical planning and determine the options for treatment and management of postoperative complications. However, data on MCA variations in the Myanmar population are scarce. To address this issue, the present study analyzed the morphology and anatomy of the MCA in fresh brain specimens from the general population in Myanmar.

## 2. Materials and Methods

The study was conducted using 100 specimens from 50 deceased patients obtained from the Police Surgeon Officer, Yangon General Hospital, Myanmar, after obtaining informed consent from next of kin. The study was approved by the Academic Committee of Anatomy, University of Medicine 1, Yangon, Myanmar. Fresh brain specimens without infectious disease or structural deformation were used for the study. The skull bone was sawed and the brain was removed as a routine postmortem procedure, followed a meticulous and accurate dissection of the pia mater to allow in situ visualization of the MCA.

The MCA was identified based on its emergence from the internal carotid artery (ICA) and entry into the lateral fissure, along which the arterial course was traced. False bifurcation/trifurcation was verified during the examination of termination patterns. The length of the main trunk (MT) was measured from the origin to the bi/trifurcation point, or from the origin to the site of the first cortical branch in cases where there was no bi/trifurcation.

The cortical branches were confirmed by tracing, and the sulcus in/over which the artery lay was noted. Cortical branches were classified as follows: orbitofrontal, directed toward the orbital surface of the frontal lobe; prefrontal, running toward the anterior edge of the limiting insular sulcus; precentral, reaching the precentral sulcus; central, reaching the central sulcus; anterior parietal, reaching the postcentral sulcus; posterior parietal, appearing on the surface of the brain at the posterior extremity of the Sylvian fissure; angular, appearing on the angular gyrus; temporopolar, directed toward the temporal pole; temporo-occipital, reaching the temporo-occipital area; and anterior temporal, middle temporal, and posterior temporal, lying between the temporopolar and temporo-occipital arteries. Any branches that spread over the superolateral surface of the cerebral hemisphere, irrespective of whether they emerged from the MT or post-bi/trifurcation divisions, were counted as cortical branches.

The perforators piercing the anterior perforated substance (APS) of the MCA were visualized by carefully lifting the MT. Two researchers independently identified and counted the branches. All of the examined structures were photographed (Cyber-shot DSC-P72 with optical zoom 25MP; Sony, Tokyo, Japan). Quantitative data were analyzed using SPSS v18.0 for Windows software (SPSS Inc., Chicago, IL, USA).

## 3. Results and Discussion

The MCA originates from the ICA, lateral to the optic chiasma and posterior to the olfactory tract. The artery then takes a lateral course, lying anterior to the optic tract and reaching below the APS to extend perforating branches to internal cerebral structures. The artery enters the lateral fissure where it bifurcates, trifurcates, or terminates as a primary trunk. Within the lateral sulcus, the artery overlies the cortical tissue of the insula and extends multiple cortical branches that spread over the convex superolateral surface of the cerebral hemisphere. In the present study, we observed the course of the MCA from its origin to the point immediately before it spreads over the superolateral surface by retracting the temporal lobe at the base. Observations included the origin, termination pattern, and length of the MT of the MCA; the number, origin, and pattern of perforators; and the presence or absence of each cortical branch and early cortical branches as well as their numbers.

**Figure 1:**
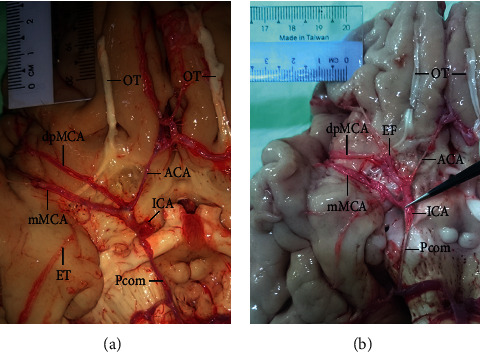
Double MCA consisting of the mMCA and the dpMCA arising from the distal part of the ICA and coexisting with hypoplasia of the anterior cerebral artery (ACA). (a) Atherosclerosis at the triple junction of the double MCA and hypoplastic ACA suggesting hemodynamic imbalance and representing a potential risk for aneurysm. (b) Origins of the dpMCA and mMCA a few millimeters apart in a location that is not associated with a high risk of hemodynamic disturbance. Early temporal (ET) and early frontal (EF) branches arising from the mMCA (a) and dpMCA (b), respectively, are shown. OT, olfactory tract; Pcom, posterior communicating artery.

The rarity of multiple MCA origins (i.e., double MCA (2%), Table 1) arising from the distal part of the ICA running parallel to the MT of the MCA and supplying the MCA territory in the Myanmar population is comparable to the frequency of 1.7–2.9% reported in other populations [11–13]. It has been proposed that double MCA, which consists of duplicated (dp) MCA and main (m) MCA [14] (Figures 1(a) and 1(b)), results from embryologic or evolutionary processes: that is, numerous twigs present in the embryo may coalesce to form a single artery, or else the dpMCA arises as a collateral branch of the anterior cerebral artery in some individuals as an evolutionary adaptation [15–17]. Duplication of the MCA at the origin can increase the risk of aneurysm formation caused by changes in hemodynamic status [18] but may also be beneficial as a collateral blood supply to the MCA territory in case of obstruction or occlusion.

**Figure 2:**
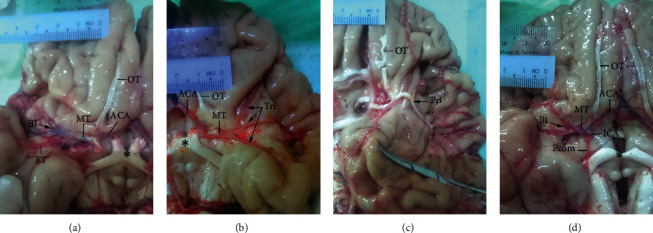
Termination patterns of the MCA. (a) The MT of the MCA terminating as a bifurcation (Bi). (b) The MT of the MCA terminating as a trifurcation (Tri). (c) Primary trunk (Pri) with severe atherosclerosis. (d) Early bifurcation resulting in a short MT just 6.5 mm in length. The ICA, anterior cerebral artery (ACA), posterior communicating artery (Pcom), early temporal branch (ET), olfactory tract (OT) (distorted in panel (c)), and optic chiasma (^*∗*^) are indicated.

**Figure 3:**
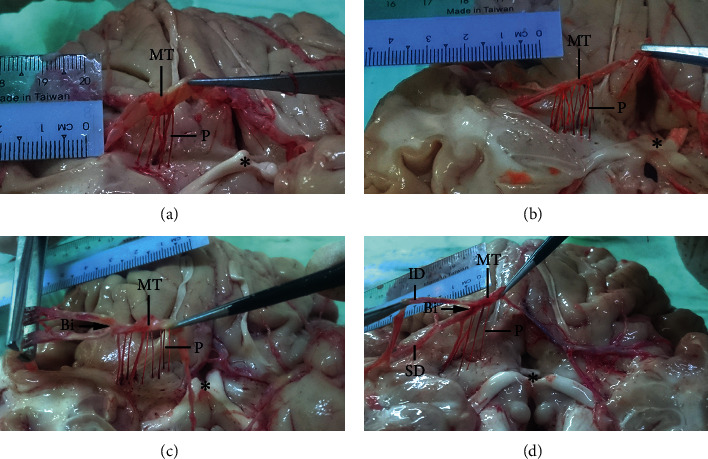
Perforators of the MCA. Six (a) and 15 (b) perforators (P) arising from the MT of the MCA as single branches. (c) Nine perforators arising from both the MT and bifurcation (Bi) point. (d) Lifting the inferior division (ID) by the forceps revealed the emergence of 5 perforators not only from the MT but also from the bifurcation and postbifurcation divisions comprising superior and inferior divisions (SD and ID, respectively). ^*∗*^Optic chiasma.

Branches sprouting from the main artery had different termination patterns (Table 1) including bifurcation (Figure 2(a)), trifurcation (Figure 2(b)), or primary trunk (Figure 2(c)); of these, bifurcation was the most common (72%), consistent with previous reports [19–24]. Early bifurcation, defined as occurring within 1 cm of the MCA origin [13], was observed in 3% (Figure 2(d) and Table 1) and should be distinguished from double MCA. Early bifurcation was more likely to be associated with perforating branches arising from the postbifurcation segment of MCA. This condition, i.e., the presence of both early bifurcation and perforators from the postbifurcation segment in the same hemisphere, was observed in 1% of specimens (Figure 3(d)). Great caution must be exercised during surgical repair of MCA aneurysms in patients with early bi/trifurcation to avoid damaging perforating branches arising from the postbifurcation segment.

The mean length of the MT of the MCA was 20.6 mm (Table 2); the length varied as previously reported [12, 20, 24]. This length is measured preoperatively to determine the optimal surgical approach for dissecting and clipping MCA bifurcation aneurysms, for which 2 surgical techniques are used, i.e., opening of the Sylvian fissure from proximal to distal (for a short MT) or from distal to proximal (for a long MT). The average length of the MT of the MCA is therefore of great interest to neurosurgeons [25].

In line with previous studies [23, 26], the number of perforators ranged from 4 to 15 with a mean number of 9.0 ± 2.6, a mode of 9, and 4 as the least frequent number (Table 2). The number of perforators was inversely related to vessel diameter (Figures 3(a) and 3(b)). The perforators arose from the MT in 96 hemispheres, from the bifurcation point in 3 hemispheres (Figure 3(c)), and from postbifurcation divisions in 1 hemisphere (Figure 3(d)). All of the perforators (100%) consisted of a single branch. The site of origin of perforating arteries is critical for neurosurgical planning. In the case of perforators arising from the bifurcation point (as observed in 3 hemispheres in the present study, Table 1), the orifice of the perforating branches may open into the mouth of the aneurysm. If endovascular coiling were used as the treatment, the neurologic consequences would be unacceptable. Additionally, MCA bifurcation aneurysms can mimic the clinical presentation of an ischemic/infarct internal capsule stroke caused by distortion or compression of perforators at the bifurcation point [27].

Intimacy with perforators and early cortical branches makes surgery on prebifurcation aneurysms of the MCA challenging. It is extremely important to establish the anatomy of the MT of the MCA and its arterial (both cortical and perforating) branches during surgical planning, as damage to these structures during dissection or occlusion of an aneurysm can give rise to neurologic sequelae. Meticulously isolating perforators, preventing vessel kinking, and selecting the most appropriate clip according to the anatomy of the MCA are essential for the successful surgical treatment of aneurysms [28].

**Figure 4:**
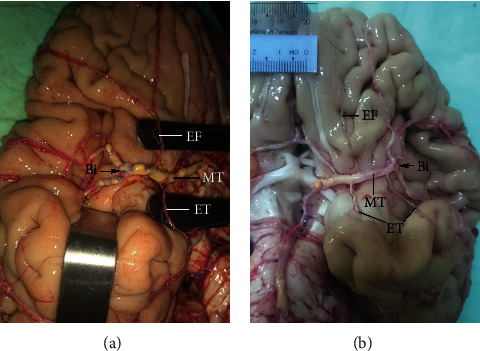
Early cortical branches. (a) Early frontal (EF) and early temporal (ET) branches. (b) Three early cortical branches (1 EF and 2 ET). The MT and bifurcation (Bi) of the MCA are indicated.

Early cortical branches arising from the MT of the MCA were observed in 52% of specimens (Table 1), which is comparable to the rates reported by others [11, 24], although one study found a much higher percentage [21]. The early frontal branches in the present study were usually orbitofrontal, and early temporal branches were usually temporopolar (Figure 4(a)) and sometimes anterior temporal (Figure 4(b)). This may be related to the intimacy of the MT of the MCA and the orbitofrontal surface and temporal pole. Of clinical note, the origin of the early cortical branch is a potential site of aneurysms; a higher frequency of early cortical branches increases aneurysm risk.

**Figure 5:**
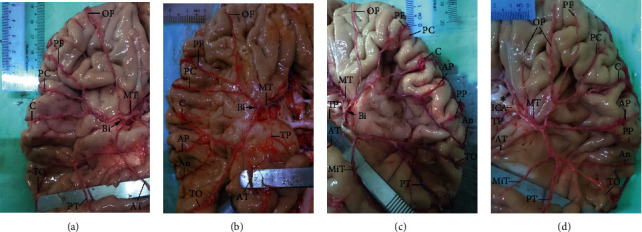
Cortical branches of the MCA. The number of cortical branches varied; 7 (a), 9 (b), 12 (c), and 13 (d) cortical branches are shown as examples. The identified cortical branches were orbitofrontal (OF), prefrontal (PF), precentral (PC), central (C), anterior parietal (AP), posterior parietal (PP), angular (An), temporopolar (TP), anterior temporal (AT), middle temporal (MiT), posterior temporal (PT), and temporo-occipital (TO). The MT and bifurcation (Bi) of the MCA and ICA are indicated. ^*∗*^Optic chiasma.

The number of cortical branches ranged from 6 to 13 (Figures 5(a)–5(d) and Table 2), reflecting early vs. late ramification of the artery during vasculogenesis, which produces more or fewer branches, respectively. The close proximity of the superior division to the frontal lobe and of the inferior division to the temporal lobe suggests higher rates of frontal lobe and temporal lobe arteries emerging from the superior and inferior divisions, respectively. However, parietal lobe arteries showed a greater dependence on the length of divisions, emerging from the superior division in cases of longer superior division (Figure 5(c)) and from the inferior division in cases of shorter inferior division (Figure 5(b)).

Frontal lobe arteries including the prefrontal, orbitofrontal, central, and precentral branches were present in >95% of specimens (Table 1), possibly reflecting the higher energy consumption of the frontal lobe for higher-order cognitive processes such as thinking, decision-making, and planning. The size and complexity of the frontal lobe distinguishes humans from other primates; it is likely that the blood supply increased concomitantly with frontal lobe expansion during primate evolution.

In most cases, the middle temporal artery did not exist as a separate branch (observed in 24% of hemispheres) (Table 1). This artery supplies the middle portion of the temporal lobe and may constitute an anastomosis between the anterior and posterior temporal arteries. If the latter 2 arteries were long enough to supply the middle portion of the temporal lobe, the middle temporal artery would not be needed and would not exist.

Temporal lobe arteries are usually used in superficial temporal cortical artery bypass operations to relieve symptoms of cerebrovascular insufficiency. An arterial branch lying on the temporal lobe is a convenient recipient in this procedure [29]. Because of their high frequencies (Table 1), the temporopolar (87%) and anterior temporal (89%) arteries may be easily located by surgeons. However, the frequency of these arteries was lower than that of frontal lobe arteries, suggesting that neural activity related to hearing and language (temporal lobe functions) consumes less energy than that associated with mental processing (frontal lobe functions), although this remains to be validated through experiments.

## 4. Conclusions

The MCA is clinically important in diseases such as stroke and intracranial aneurysms. Preoperative angiographic examination is performed to evaluate the anatomy of the MCA. Anatomic variations of the MCA can affect surgical decisions; moreover, they may be detected incidentally later by radiography and misinterpreted as abnormal findings. Thus, data pertaining to such variations in the Myanmar population are needed to improve surgical planning and outcomes. Our observations from autopsy specimens provide accurate and valuable information for radiologists, anatomists, and neurosurgeons.

## Figures and Tables

**Table 1:** Main trunk, perforators, and cortical branches of the MCA.

	R	L	Total
**MT of the MCA**			
*Origin*			
Double MCA	2	−	2
Usual origin	48	50	98

*Termination pattern*			
Bifurcation	33	39	72
Trifurcation	11	5	16
Primary trunk	6	6	12

*Early bi/trifurcation (based on 72% and 16% bi/trifurcation)*			
Present	1	2	3
Absent	43	42	85

**Perforators of the MCA**			
*Origin*			
MT only	47	49	96
MT and bifurcation	2	1	3
MT, bifurcation, and postbifurcation	1	−	1

*Patterns*			
As single branches	50	50	100
As a common trunk	−	−	−

**Cortical branches**			
*Frontal lobe arteries*			
Orbitofrontal	50	48	98
Prefrontal	50	49	99
Precentral	49	46	95
Central	49	49	98

*Temporal lobe arteries*			
Temporopolar	43	44	87
Anterior temporal	43	46	89
Middle temporal	11	13	24
Posterior temporal	32	30	62
Temporo-occipital	36	33	69

*Parietal lobe arteries*			
Anterior parietal	45	43	88
Angular	40	43	83
Posterior parietal	31	26	57

*Early cortical branches*			
Early frontal branch only	3	2	5
Early temporal branch only	20	20	40
Both early frontal and temporal branches	4	3	7
Early parietal branch	−	−	−

Data are expressed as a percentage of 100 hemispheres. Abbreviations: L, left; MCA, middle cerebral artery; MT, main trunk; R, right.

**Table 2:** Length of the main trunk of the MCA and number of perforators and cortical branches.

*Length of main trunk of the MCA (mm)*	
Mean ± SD	20.6 ± 6.2
Range	6.5–35.0

*Number of perforators*	
Mean ± SD	9.0 ± 2.6
Range	4–15
Mode	9
Least frequent number	4

*Number of cortical branches*	
Mean ± SD	9.3 ± 1.3
Range	6–13
Mode	9
Least frequent number	6 and 13

Abbreviations: MCA, middle cerebral artery; SD, standard deviation.

## Data Availability

The data used to support the findings of this study are included within the article.
